# Pica for Uncooked Basmati Rice in Two Women with Iron Deficiency and a Review of Ryzophagia

**DOI:** 10.1155/2016/8159302

**Published:** 2016-01-04

**Authors:** James C. Barton, J. Clayborn Barton, Luigi F. Bertoli

**Affiliations:** ^1^Southern Iron Disorders Center, Birmingham, AL 35209, USA; ^2^Department of Medicine, University of Alabama at Birmingham, Birmingham, AL 35294, USA; ^3^Department of Medicine, Brookwood Medical Center, Birmingham, AL 35209, USA; ^4^Brookwood Biomedical, Birmingham, AL 35209, USA

## Abstract

Reports of pica for uncooked rice (ryzophagia) in adults who reside in European and derivative countries are uncommon. We evaluated and treated two nonpregnant women with pica for uncooked basmati rice. Both women reported fatigue, abdominal discomfort after consuming large quantities of uncooked basmati rice, and hair loss. One woman was from India and the other was from Pakistan. Both women were vegetarians. Basmati was the local rice in their native countries and their usual rice in the USA. Both women had tooth damage due to eating uncooked rice and iron deficiency with microcytic anemia attributed to menorrhagia and multiparity. Ryzophagia and other manifestations (except tooth damage) resolved after iron dextran therapy. We review and discuss other reports of ryzophagia associated with iron deficiency, pregnancy, race/ethnicity, geographic origin, and local traditions. We conclude that adults with ryzophagia in European and derivative countries are likely to be non-Europeans.

## 1. Introduction

Pica is the compulsive, repetitive eating of nonfood substances or large amounts of specific foods or condiments daily for more than one month [1–4]. In adults, pica for items not part of one's habitual diet or preferences is a common but incompletely understood accompaniment of iron deficiency [5–9] and pregnancy [10–14]. Pica items in adults with iron deficiency are diverse, typically contain little or no absorbable iron, and vary according to personal choice, race/ethnicity, culture, item availability and convenience, and geographic location [15–19]. Although pica for uncooked rice (ryzophagia) has been reported in nonpregnant adolescents and adults with iron deficiency [19, 20] and in pregnancy [21], other aspects of ryzophagia are undescribed.

We report the evaluation and treatment of two women with iron deficiency, one from India and the other from Pakistan, who developed pica for uncooked basmati rice. We discuss particular aspects of basmati ryzophagia in the context of the two present women and review ryzophagia in adults of European and non-European ancestry and its relationships to iron deficiency, pregnancy, race/ethnicity, local traditions, geographic origin, and other conditions.

## 2. Case Reports

*Patient 1*. A 39-year-old woman, a native of Mumbai, India, presented with severe fatigue and hair loss. She reported pica for uncooked milled basmati rice, the predominant local rice in Mumbai and her usual rice in the USA. This manifestation had occurred for many years and was more intense during pregnancy. She often developed abdominal pain after eating large quantities of uncooked rice. She was a vegetarian. She reported that her mother, grandmother, and other women in Mumbai also craved uncooked basmati rice. Physical examination revealed moderate pallor and several teeth chipped by eating uncooked rice. Her iron deficiency and microcytic anemia (Table 1) were attributed to menorrhagia and multiparity. She was intolerant of oral iron supplements and thus was treated with intravenous iron (1500 mg Fe, total) [18]. Pica resolved after the first infusion of 500 mg of iron [18]. Other manifestations (except tooth damage) resolved after completion of iron dextran therapy.

*Patient 2*. A 44-year-old woman, a native of Karachi, Pakistan, presented with fatigue, dizziness, cold intolerance, hair loss, and decreased growth and thinning of nails. She reported having pica for uncooked milled basmati rice (the most popular rice in Karachi and her usual rice in the USA), drying cement, and dirt. She often developed abdominal pain after eating large amounts of uncooked basmati rice. She was a vegetarian. Physical examination revealed moderate pallor and chipped and abraded teeth due to eating her pica substances. Iron deficiency and microcytic anemia (Table 1) were attributed to menorrhagia and multiparity. She was intolerant of oral iron supplements and thus was treated with intravenous iron (1000 mg total) [18]. Pica resolved after the first infusion of 500 mg of iron [18]. Other manifestations (except tooth damage) resolved after completion of iron dextran therapy.

## 3. Literature Search

We performed a computerized search of the National Library of Medicine (http://www.pubmed.gov) and other internet sites to identify reports of ryzophagia (rizophagia). In some studies, pica for uncooked rice (ryzophagia) is categorized as a subtype of starch pica (amylophagia, amylophagy) [21, 22]. Thus, we used amylophagia as an additional search term. We also performed a manual search of many reports of iron deficiency, pregnancy, pica, and amylophagia to identify other cases of ryzophagia.

## 4. Discussion

The present women had iron deficiency complicated by microcytic anemia, fatigue, hair and nail symptoms, and pica for uncooked milled basmati rice. These observations are consistent with previous reports of ryzophagia in men and nonpregnant women. In 16 adolescent girls with anemia or iron deficiency on Réunion Island (La Réunion), 81% had pica for uncooked rice [20]. In 79 European and non-European patients (92% women) with iron deficiency in France, 4% had pica for uncooked rice [19]. None of 79 control subjects reported ryzophagia [19].

Pica for uncooked basmati rice was more intense during pregnancy in Patient 1. In 2,367 pregnant women on Pemba Island, Zanzibar, Tanzania, 897 had pica [21]. Of those 897, 86% had pica for uncooked rice alone and 4% had pica for both uncooked rice and earth [21]. In comparison with pregnant women without pica, women who routinely ate uncooked rice had mean hemoglobin concentrations that were 0.6 g/dL lower (*P* < 0.001) and those who consumed uncooked rice and earth had mean Hb concentrations that were 1.1 g/dL lower (*P* < 0.001). A pregnant woman in India without report of iron measures also had pica for uncooked rice and wheat [23].

Abdominal discomfort or pain occurred in the present patients after they ate large quantities of uncooked basmati rice. In pregnant women on Pemba Island, the prevalence of nausea and abdominal pain was significantly greater among those who had consumed uncooked rice during pregnancy (mean daily consumption 34.5 g (range 7.7–77.9 g) or ~1/3 cup, on average) than those who had not consumed uncooked rice (*P* < 0.05) [21]. Abdominal discomfort or pain was also reported by another pregnant woman with ryzophagia [23] and by other patients with iron deficiency who had either arabositophagia [24] or geophagia [25]. Tooth damage occurred in both of the present women, in another woman who ate uncooked rice [23], and in other persons with iron deficiency and pagophagia [5]. Both of the present women reported hair loss. Iron deficiency was significantly associated with hair loss in three studies [26–28] but not in another report [29].

Mean corpuscular volume (MCV), a surrogate measure of tissue iron stores [30], is inversely associated with pica in persons with iron depletion or deficiency [6, 8]. This is consistent with the present observations and with pretreatment laboratory test results in nine adolescent females with pica for uncooked rice and iron deficiency on Réunion Island (mean MCV 65.9 ± 8.3 (SD) fL) [20]. Iron deficiency and its severity were independent predictors of pica in adults in regression analyses [6, 19]. Pica in the present women resolved after they were treated for iron deficiency, in agreement with other reports [6, 19, 20]. Recurrence of pica is often a harbinger of recurrent iron deficiency [5, 6]. These observations infer that iron depletion or deficiency is causally related to pica in some adults, although the biochemical mechanism(s) by which iron depletion or deficiency induces pica remains obscure.

It is plausible that the predominant cause of pica associated with iron deficiency or depletion in adults is low tissue iron levels, possibly in the tongue, olfactory apparatus, or other locations in the brain [31, 32]. In iron-deficient rats, mean blood ^59^Fe levels after intranasal administration of the radioisotope as ferrous or ferric form were significantly higher than those of iron-sufficient control rats [33]. Divalent metal transporter-1 (DMT1) levels are significantly higher in the olfactory bulbs of iron-deficient rats [33]. Thus, the molecular mechanism of olfactory iron absorption and possibly of olfactory or gustatory function involves DMT1 and is influenced by body iron repletion. The transport of noniron divalent metal ions such as manganese via nasal mucosa is also increased in iron-deficient mice [34] and may contribute to pica. It has been suggested that the iron content of the hippocampus influences the expression of pica in humans [35]. Hunt et al. demonstrated that chewing ice (pagophagia) significantly improved response time on a neuropsychological test among persons with iron-deficiency anemia but not in control subjects [36]. Potential explanations for these observations include activation of the dive reflex, which would lead to peripheral vasoconstriction and preferential perfusion of the brain or, alternatively, sympathetic nervous system activation, which would also increase blood-flow to the brain [36]. Other factors driving pica in adults with iron deficiency are related to geographic location, race/ethnicity, cultural attributes, available pica items, age, and gender [6, 37]. Gross epithelial manifestations of iron deficiency or depletion such as glossitis or cheilosis or common alleles of the* TMPRSS6* gene that encodes matriptase-2, a serine protease that represses hepcidin, probably do not cause or influence pica [6, 38].

The iron content of rice grains, estimated using chemical analyses or Prussian blue staining [39, 40], varies according to cultivar, region of cultivation, and other factors, including genetic manipulation [39–41]. Milling removes much of the iron from rice grains [40]. In previous reports of ryzophagia, it was inferred but not explicitly stated that the rice was milled [19–21, 23]. No study has assessed the bioavailability of micronutrients in uncooked rice, but it is unlikely that more nutrients would be available in uncooked than cooked rice [21]. The “white” rice consumed in Zanzibar is not enriched and has low levels of most micronutrients [42]. Bioavailability of iron in meals that contain cooked rice may be reduced by the phytate content of the rice grains [43, 44].

Basmati ryzophagia in the two present women may have been triggered in part by the aromatic properties of the rice. The predominant aromatic compound in basmati rice, 2-acetyl-1-pyrroline, accounts for its popcorn-like smell [45, 46]. Brown and milled basmati rice retain this key aroma and flavor compound [46, 47]. 2-Acetyl-1-pyrroline is also an important aroma and flavor compound in popcorn [45, 48], pica for which (arabositophagia) has been reported in patients of European descent with iron deficiency [6, 24]. The type(s) of uncooked rice consumed by patients in previous reports [19–21, 23] was not specified. In Malagasy people, smell motivated geophagia in 5% of study subjects and consumption of other pica items in 4% [22]. In response to craving, some pregnant women smell particular substances alone or with pica [31, 32]. Rats with iron deficiency had longer exploratory times for attractive odorants than control rats [33].

Both of the present women were vegetarians and non-Europeans. Kettaneh et al. performed a case-control study of 79 Europeans and non-Europeans with iron deficiency and 79 controls, all of whom resided in France [19]. In a univariable comparison, 33% (7/21) of vegetarians had pica, whereas only 7% (4/58) of nonvegetarians had pica (*P* = 0.006) [19]. In a logistic regression, iron-deficiency anemia and being non-European were significant independent predictors of pica [19]. Ryzophagia was reported by 4% of the patients with iron deficiency, although whether patients with ryzophagia were Europeans or non-Europeans was not reported [19].

Six of 13 iron-deficient adolescent females on Réunion Island who reported pica for uncooked rice also had pagophagia (46%) [20]. Two (15%) also had geophagia, like Patient 2. Polypica is relatively common in large case series of persons with iron depletion or deficiency [5, 6, 19]. Ice is a common or the predominant pica item in case series of whites of European descent, South African blacks, and African Americans with iron deficiency [5, 18, 19, 49–51]. In a study of pregnant women on Pemba Island, Young et al. excluded ice from pica analyses because the study subjects had infrequent access to ice [21]. Geophagia was the predominant pica item in one study of black patients with iron deficiency in South Africa [5]. Pregnant Saudi women with iron deficiency craved milk, salty and sour foods, sweets, and dates [17]. These observations substantiate that no pica item is specific for iron deficiency or depletion. The items vary according to personal choice, race/ethnicity, culture, availability and convenience, and geographic origin of patients [15–19].

Regular consumption of uncooked rice was not regarded as pica in one study [22]. In a population-based interview study of persons >5 years of age in 16 villages in the Makira Protected Area of Madagascar, the prevalence of geophagia was 53%, of consumption of raw starchy items was 85%, and of other unusual substances was 19% [22]. The starchy items were raw cassava, raw sweet potato, uncooked rice, and* ambaradedin-ambazaha* (genus* Hedychium*, a wild root vegetable which is consumed raw) [22]. The proportion of men who consumed these items was high (>90%). The proportion of women who consumed these items was not higher during pregnancy, although observations on only 4 pregnant women were reported [22]. Pulverized rice husks were consumed by 6% of the 760 study subjects [22]. Golden et al. concluded that all of these substances were consumed as self-medication or food, especially the starchy items, but not as pica [22]. On the other hand, these 760 subjects were not evaluated for anemia or iron status. In a study of food cravings (not defined as pica) reported by 185 female undergraduate students in Japan, cooked rice was the most popular item [52]. Komatsu concluded that “rice craving” may be characteristic of Asian rice-consuming countries and that craving for some foods is influenced by traditions of food products and cultures [52].

Uncertainties of our literature review include the possibility that we did not identify some reports of ryzophagia in our computerized searches because the reports were not indexed with search terms we used. We may have overlooked reports of ryzophagia in manual searches because it was not feasible to review all published reports of pica.

## 5. Conclusions

We conclude that adults with ryzophagia in European and derivative countries are likely to be non-Europeans. It is plausible but unproven that ryzophagia in adults is a common but infrequently reported manifestation of iron deficiency or pregnancy in large regions of Asia and sub-Saharan Africa where rice is a major dietary staple [53, 54], vegetarianism is common [55–57], and the prevalence of iron deficiency in the general population is high [58, 59]. Physicians should inquire about pica at diagnosis of iron deficiency or depletion in all adults. The prevalence of ryzophagia may be greater in non-Europeans, especially women. Evaluation to identify causes of iron deficiency or depletion, possible dental trauma, and complaints of abdominal discomfort may also be appropriate in such patients.

## Figures and Tables

**Table 1:** Laboratory results in two women at diagnosis of iron deficiency^1^.

Analyte	Patient 1	Patient 2
Hb, g/dL	8.2	9.5
RBC × 10^6^/*µ*L	4.44	3.83
MCV, fL	61.0	79.8
RDW, %	18.1	15.0
WBC × 10^3^/*µ*L	5.1	6.6
Platelets × 10^3^/*µ*L	239	324
Serum iron, *µ*g/dL	25	2
TS, %	5	5
Serum ferritin, *µ*g/L	8	5

^1^Iron deficiency was defined as both TS <10% and SF <20 *µ*g/L [6]. Hb: hemoglobin; RBC: red blood cells; MCV: mean corpuscular volume; RDW: red blood cell distribution width; WBC: white blood cells; TS: transferrin saturation.
